# First record of *Tettigettalna mariae* Quartau & Boulard, 1995 (Insecta: Hemiptera: Cicadoidea) in Spain

**DOI:** 10.3897/BDJ.1.e978

**Published:** 2013-09-16

**Authors:** Paula C. Simões, Vera L. Nunes, Raquel Mendes, José A. Quartau

**Affiliations:** † Faculdade de Ciências da Universidade de Lisboa, Departamento de Biologia Animal e Centro de Biologia Ambiental, Computational Biology and Population Genomics Group, Edifício C2, Piso 3, Campo Grande, 1749-016 Lisboa, Portugal, Lisbon, Portugal

**Keywords:** Cicada, first record, *Tettigettalna mariae*, Spain

## Abstract

*Tettigettalna mariae* Quartau & Boulard 1995 is recorded for the first time in Spain. Thought to be endemic to Portugal (occurring in the southern province of Algarve), the present paper adds its distribution to southern Spain, being an Iberian endemism. The acoustic signals of the new specimens collected were recorded in different localities of Huelva province, in Andalusia during August 2012. According to their present known distribution, specimens of *Tettigettalna mariae* tend to be sparsely distributed in small range populations in southern Iberian Peninsula, favouring wooded areas with *Pinus pinea*.

## Introduction

Cicadas (Hemiptera: Cicadoidea) constitute a successful group of insects where males typically communicate during pair formation and courtship through acoustic distinctive signals (Claridge 1985, Boulard and Mondon 1995, Simões et al. 2000, Quartau et al. 1999). Despite being common and with several thousands of species described worldwide, cicada’s biodiversity is still poorly known. Recent work has drawn some concern to the diversity of species in the Iberian Peninsula (Boulard 1982, Boulard 2000, Puissant and Sueur 2010, Sueur et al. 2004, Quartau and Fonseca 1988), showing that this region is an important hotspot for Mediterranean cicada diversity. Particular attention was given recently to smaller species in southern Iberia with nine species assigned to the genus *Tettigettalna*, seven of which are believed to be endemic to southern Iberia (Puissant and Sueur 2010). Three species (*Tettigettalna mariae*, *Tettigettalna josei* and *Tettigettalna estrellae*) have been described and recorded so far in Portugal only (Sueur et al. 2004) but their distribution range in other parts of the Iberia Peninsula has never been investigated before.

The current knowledge on the distribution boundaries of *Tettigettalna* species is far from being properly known and extensive field surveys for these cicadas are still missing. Moreover, some of these species are believed to have very restricted distribution ranges as is the case of *Tettigettalna mariae*, a new species that was recently described (Quartau and Boulard 1995) and was thought to be endemic to the Algarve, the southern province of Portugal.

## Materials and methods

As an outcome of intensive fieldwork in Portugal and Spain for species of the genus *Tettigettalna* during the summer of 2012, we report here the discovery of a new populations of *Tettigettalna mariae* in Andalusia (Southern Spain). Identification was based on the collection of specimens and the recording of male acoustic signals typical of the species.

The field survey was conducted daily from 10:00 h to 19:00 h during sunny weather with temperatures ranging from 29° to 32° C. Searches were conducted by car while driving at a reduced speed, allowing the detection of the songs of calling males. Their acoustic signals were recorded at the collecting site using a Marantz PMD 661 Portable SD recorder (20 Hz – 24 kHz) connected to a Telinga Pro 7 Dat-mic microphone (Twin Science) following the procedures given in Simões et al. 2000. Specimens were then captured by hand or by means of a sweeping net.

Geographical coordinates were determined with a GPS (Garmin, Oregon series 550t) for each site where male songs were heard and where specimens were collected. Species confirmation was accomplished with time and frequency analysis of sound recordings using the software Avisoft Sas-Lab Pro (Specht 2012) as in previous analyses (e.g. Quartau et al. 1999, Simões et al. 2000). Acoustic recordings were analyzed with a sampling rate of 44.1 kHz and a resolution of 16 bits. Spectra were computed using FFT with a resolution of 512 points and a Hamming Window. For each male, recordings of about one minute were analysed. Song terminology follows that of Gogala and Trilar (1999) and Gogala and Trilar (2000). The examined material and acoustic recordings are deposited in the general data bank on insect data at the Department of Animal Biology in the Faculty of Sciences, University of Lisbon (FCUL). Collected specimens were stored dry and a front leg was preserved in 100% ethanol for DNA isolation.

## Taxon treatments

### Tettigetalna mariae

Quartau & Boulard, 1995

#### Materials

- **Type status:** Other material. **Occurrence:** recordedBy: Raquel Mendes; individualCount: 3; sex: male; **Location:** country: Espana; stateProvince: Andaluzia; verbatimLocality: Cartaya; verbatimLatitude: 37°15'44.2"N; verbatimLongitude: 7°07'48.9"W; **Event:** samplingProtocol: Acoustic recording; eventDate: 2012-08-15T17:00Z; **Record Level:** collectionID: 3372;3373;3374; institutionCode: FCUL; collectionCode: Entomology_PCS
- **Type status:** Other material. **Occurrence:** recordedBy: Raquel Mendes; Vera Nunes; individualCount: 3; sex: male; **Location:** country: Espana; stateProvince: Andaluzia; verbatimLocality: Cartaya; verbatimLatitude: 37°15'44.2"N; verbatimLongitude: 7°07'48.9"W; **Event:** samplingProtocol: Acoustic recording; eventDate: 2012-08-15T18:00Z; **Record Level:** collectionID: 3375;3376;3377; institutionCode: FCUL; collectionCode: Entomology_PCS
- **Type status:** Other material. **Occurrence:** recordedBy: Vera Nunes; individualCount: 1; sex: male; **Location:** country: Espana; stateProvince: Andaluzia; verbatimLocality: Cartaya; verbatimLatitude: 37°15'44.2"N; verbatimLongitude: 7°07'48.9"W; **Event:** samplingProtocol: Sweep net capture; eventDate: 2012-08-15T17:45Z; **Record Level:** collectionID: 3378; institutionCode: FCUL; collectionCode: Entomology_PCS
- **Type status:** Other material. **Occurrence:** recordedBy: Raquel Mendes; Vera Nunes; individualCount: 1; sex: male; **Location:** country: Espana; stateProvince: Andaluzia; verbatimLocality: Moguer; verbatimLatitude: 37°12'30.7"N; verbatimLongitude: 6°46'46.1"W; **Event:** samplingProtocol: Acoustic recording; eventDate: 2012-08-16T11:50Z; **Record Level:** collectionID: 3379; institutionCode: FCUL; collectionCode: Entomology_PCS
- **Type status:** Other material. **Occurrence:** recordedBy: Raquel Mendes; Vera Nunes; individualCount: 1; sex: male; **Location:** country: Espana; stateProvince: Andaluzia; verbatimLocality: Moguer; verbatimLatitude: 37°12'30.7"N; verbatimLongitude: 6°46'46.1"W; **Event:** samplingProtocol: Acoustic recording; eventDate: 2012-08-16T14:40Z; **Record Level:** collectionID: 3380; institutionCode: FCUL; collectionCode: Entomology_PCS
- **Type status:** Other material. **Occurrence:** recordedBy: Vera Nunes; individualCount: 1; sex: male; **Location:** country: Espana; stateProvince: Andaluzia; verbatimLocality: Almonte; verbatimLatitude: 37°13'43.0"N; verbatimLongitude: 6°33'51.1"W; **Event:** samplingProtocol: Acoustic recording; eventDate: 2012-08-16T15:45Z; **Record Level:** collectionID: 3382; institutionCode: FCUL; collectionCode: Entomology_PCS

## Analysis

Our analysis of the morphological and acoustic data confirmed the presence of *Tettigettalna mariae* specimens in Spain.

**Figure 1.**
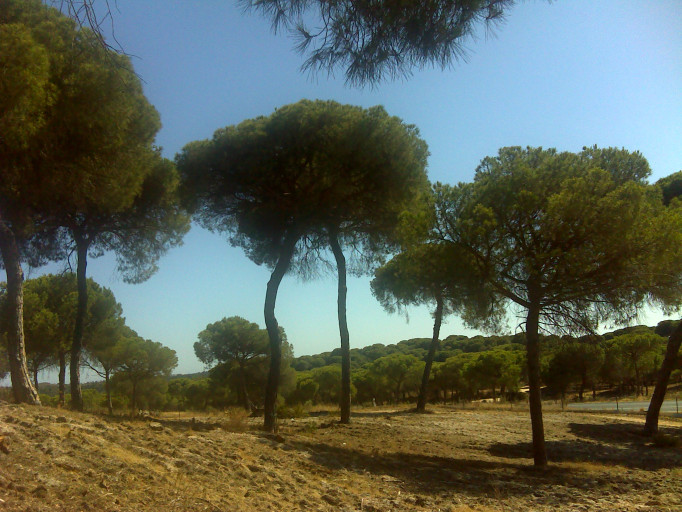
Habitat of *Tettigettalna mariae* in Cartaya (Huelva, Spain) corresponding to a typical wooded area of *Pinus pinea*. Males were usually singing on pine branches or leaves.

**Figure 2.**
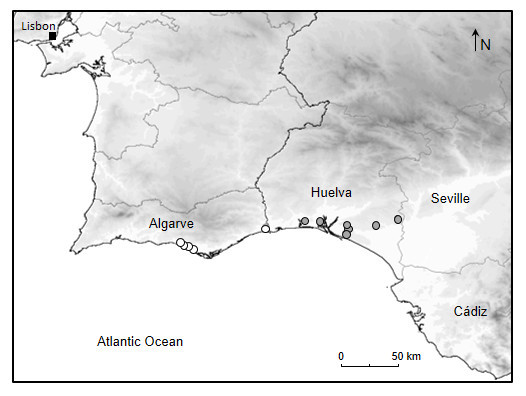
Map of occurrence of *Tettigettalna mariae* in the south of the Iberian Peninsula, showing former documented populations in Algarve (Portugal) according to Sueur *et al.* (2004) (white circles) and the new populations recorded in August 2012 (grey circles) in the province of Huelva, Andalusia (Spain).

Specimens were collected and recorded in different locations from Huelva province in Andalusia around the following localities: Cartaya, Aljaraque, Moguer, Mazagón, Almonte and Hinojos (Table 1). Records were sparse, even within large patches of suitable habitat, and match its current habitat preference, with *Tettigettalna mariae* tending to favour wooded areas of *Pinus pinea* near the sea in the southern Iberian Peninsula (Figs 1, 2).

**Figure 3.**
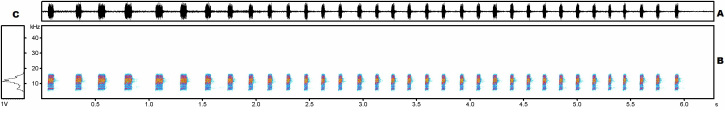
Calling song profile of a *Tettigettalna mariae* specimen recorded in Cartaya (Huelva, Spain). A - Oscillogram (amplitude *vs*. time), B - sonagram or spectrogram (frequency *vs*. time) and C - mean amplitude spectrum (frequency *vs*. amplitude).

Acoustic analysis (Table 2, Fig. 3) showed the profile of the calling song in agreement with previous studies (Quartau and Boulard 1995, Fonseca 1991, unpublished data). *Tettigettalna mariae* specimens have a broad spectrum near 6 - 16.5 kHz with maximum energy around 12 kHz. For time domain variables, our results indicated an echeme duration ranging from 0.02 to 0.10s, with an average value of 0.06s. For the echeme period we found a range of 0.16 to 0.54s and average of 0.32s.

## Discussion

Previous studies suggested that *Tettigettalna mariae* was a Portuguese endemic cicada, seeming to be confined to central Algarve, close to the sea (Sueur et al. 2004) which is an area under increasing human pressure. The coastline of Algarve is heavily urbanized, with many touristic villages and golf courses covering most of Vilamoura, Vale do Lobo and Quinta do Lago. This raises concerns about the conservation of *Tettigettalna mariae* given the restricted habitat range of the known populations. The discovery of *Tettigettalna mariae* populations in Spain means that the species is not confined to the central wooded area of Algarve, close to the sea, having instead a wider distribution extending to Andalusia. The new populations of *Tettigettalna mariae* reported here constitute an important addition to the scarce knowledge of this rare species. However, *Tettigettalna mariae* distribution remains heavily fragmented and discontinuous. Consequently the species is still vulnerable to habitat loss caused by changes in land use or forest fires that often jeopardize *Pinus pinea* woods during the summer, when cicada adult males are active. These threats may cause the decline and eventual extinction of local populations of cicadas and are especially worrying for small range species such as *Tettigettalna mariae* (Quartau and Mathias 2010).

With the present data, obtained through our 2012 fieldwork, a new cicada species is listed for Spain and a new endemism for Iberia.

Moreover, the current species list available for the cicadas from Iberian Peninsula are likely to still be incomplete. As the male acoustic signals in cicadas are highly diagnostic for the separation of closely related species (Claridge 1985, Boulard and Mondon 1995, Gogala et al. 2008, Gogala et al. 2011), it is quite possible that to the same specific name may correspond in fact two or more independent sibling species as has happened in other genera, such as *Cicadetta* (*e.g.*Gogala et al. 2008, Gogala et al. 2011). All this suggests the presence of a larger number of species in the Iberian Peninsula than those already recorded and calls for further cicada surveys in the area, as well as a better knowledge of cicada biology and ecology, which is the key to the conservation of these interesting insects in the Mediterranean area.

## Supplementary Material

XML Treatment for Tettigetalna mariae

## Figures and Tables

**Table 1.** List of localities from the province of Huelva (Andalusia, Spain) where specimens of *Tettigettalna mariae* were detected. Type of observation: Ao = Audio only (sound heard but not recorded), Ar = Audio recording and C = Captured.

Locality	GPS coordinates (degrees minutes seconds)	Date	Type of observation	Specimen ID code	DNA sample code
Cartaya	37°15'44.2"N, 7°07'48.9"W	15/08/2012	Ar	3372	__
Cartaya	37°15'44.2"N, 7°07'48.9"W	15/08/2012	Ar	3373	__
Cartaya	37°15'44.2"N, 7°07'48.9"W	15/08/2012	Ar	3374	__
Cartaya	37°15'44.2"N, 7°07'48.9"W	15/08/2012	Ar	3375	__
Cartaya	37°15'44.2"N, 7°07'48.9"W	15/08/2012	Ar	3376	__
Cartaya	37°15'44.2"N, 7°07'48.9"W	15/08/2012	Ar	3377	__
Cartaya	37°15'44.2"N, 7°07'48.9"W	15/08/2012	C	3378	Tma3378
Aljaraque	37°15'50.1"N, 7°00'29.6"W	17/08/2012	Ao	__	__
Moguer	37°13'55.2"N, 6°47'48.7"W	16/08/2012	Ao	__	__
Moguer	37°12'30.7"N, 6°46'46.1"W	16/08/2012	Ar	3379	__
Moguer	37°12'30.7"N, 6°46'46.1"W	16/08/2012	Ar	3380	__
Mazagón	37°09'57.4"N, 6°48'23.8"W	16/08/2012	Ao	__	__
Almonte	37°13'43.0"N, 6°33'51.1"W	16/08/2012	Ar	3382	__
Hinojos	37°16'59.4"N, 6°23'36.1"W	16/08/2012	Ao	__	__

**Table 2.** Descriptive statistics of the acoustic variables for *Tettigettalna mariae* specimens. Time variables in seconds and frequency variables in Hz.

	Ech/s	Echeme duration	Inter-echeme interval	Eheme period	Echeme rate	Peak frequency	Minimum frequency	Maximum frequency
Average	4.41	0.06	0.26	0.32	0.27	12049	5808	16380
Minimum	1.86	0.02	0.14	0.16	0.14	11569	5195	15860
Maximum	8.98	0.10	0.46	0.54	0.42	12411	7741	17244

## Supplementary files

### Tettigettalna mariae calling song

**Authors:** Raquel Mendes; Vera Nunes **Data type:** sound file Calling song of a *Tettigettalna mariae* specimen recorded in Cartaya (Huelva, Spain). **File:** Tmariae.mp3
